# Metagenomics reveals diverse community of putative mercury methylators across different biogeochemical niches in Sansha Yongle blue hole

**DOI:** 10.1007/s42995-025-00332-7

**Published:** 2025-11-19

**Authors:** Heyu Lin, Xiao-Yu Zhu, Chun-Xu Xue, Peng Yao, Liang Fu, Zuosheng Yang, Xiao-Hua Zhang, John W. Moreau

**Affiliations:** 1 School of Geographical, Atmospheric and Earth Sciences, The University of Melbourne, Parkville, VIC 3010 Australia; 2 College of Marine Life Sciences, and Institute of Evolution & Marine Biodiversity, Ocean University of China, Qingdao, 266003 China; 3 Laboratory for Marine Ecology and Environmental Science, National Laboratory for Marine Science and Technology (Qingdao), Qingdao, 266237 China; 4 Frontiers Science Center for Deep Ocean Multispheres and Earth System, and Key Laboratory of Marine Chemistry Theory and Technology, Ministry of Education, Ocean University of China, Qingdao, 266100 China; 5 Sansha Track Ocean Coral Reef Conservation Research Institute, Sansha, 573199 China; 6 College of Marine Geosciences, Ocean University of China, Qingdao, 266100 China; 7 School of Geographical and Earth Sciences, University of Glasgow, Glasgow, G12 8RZ UK; 8 Present Address: Centre for Microbiome Research, School of Biomedical Sciences, Queensland University of Technology, Translational Research Institute, Woolloongabba, QLD 4102 Australia

**Keywords:** Mercury methylation, *Hgc* genes, Blue hole, Redox gradient, Particle-associated, Free-living, Metagenomics

## Abstract

Methylmercury (MeHg) is a potent neurotoxin and bioaccumulates in food webs. Microbial transformation of inorganic mercury (Hg) produces most of the MeHg in the marine environment. The gene pair *hgcAB* encodes for Hg methylation, a process predominantly attributed to anaerobic bacteria. However, recent studies indicate the formation of methylmercury in low-oxygen zones within marine water columns, although the mechanisms remain poorly understood. “Blue holes” are marine sinkholes containing redox gradients stratified with depth and high microbial diversity across a range of biogeochemical cycles. Here, we present the first metagenomic analysis focused on the potential for Hg methylation in a blue hole ecosystem. Yongle Blue Hole (YBH), currently the world’s deepest known blue hole, was selected as a representative site to investigate the genetic potential for Hg methylation and to explore the functional capabilities of putative Hg-methylators within this unique environment. Metagenomic analysis showed that the anoxic sulfidic deep water was likely to be a hotspot for Hg methylation, driven by abundant and diverse Deltaproteobacteria. In the suboxic intermediate layer, *Nitrospina* and Myxococcota dominated the Hg-methylating community. Furthermore, Hg methylators were found to have different lifestyles (free-living or particle-associated) and to occupy distinct ecological niches within the YBH. In addition, the contribution of sinking particles to Hg methylation, especially in the deep anoxic water column, was highlighted. Our study unveils the biodiversity and survival strategies of Hg methylators across distinct environments. The findings suggest that blue holes could serve as model stratified ecosystems for studying Hg methylation processes across different habitats.

**Supplementary Information:**

The online version contains supplementary material available at https://doi.org/10.1007/s42995-025-00332-7.

## Introduction

Mercury (Hg) is a pollutant released from both natural and anthropogenic sources (Beckers and Rinklebe 2017; Boening 2000). Methylmercury (MeHg), as one of the most toxic forms of Hg, bioaccumulates in food webs (Driscoll et al. 2013). MeHg poisoning in humans has been reported in many countries throughout history, with the most notorious case in Minamata (Japan) in the 1950s, resulting in the deaths of hundreds of people (Almeida and Stearns 1998). A better understanding of the MeHg production mechanism and source is crucial to protect human health and marine food webs from exposure to MeHg contamination.

MeHg is primarily produced through a microbially-mediated process and requires the involvement of the gene pair *hgcAB* (Parks et al. 2013; Wang et al. 2024). These genes almost always appear in tandem within Hg-methylator genomes and are vital for Hg methylation, as their deletion leads to a loss of this function (Smith et al. 2015). The *hgcA* gene encodes a corrinoid iron-sulfur protein with a highly conserved [N(V/I)WC(A/S)(A/G)GK] motif crucial for coordinating its iron-sulfur cluster, and binding to a cobalamin (vitamin B12) ligand necessary for methyl group transfer. The *hgcB* gene encodes a relatively short ferredoxin-like protein, typically less than 100 amino acids in length, characterized by a series of conserved cysteine residues that form two tandem CX_2_CX_2_CX_3_C motifs. These motifs facilitate electron transfer, supporting the activity of HgcA (Lin et al. 2023; Parks et al. 2013). Despite the importance of both genes, the *hgcA* gene is more consistently used as a marker for mercury-methylating microorganisms because the short length of *hgcB* makes it challenging to identify through sequence similarity searches. Recently, international collaboration efforts have been made to collect *hgcAB* sequences from public databases and compile a database of Hg-methylating genes—Hg-MATE-Db (Capo et al. 2022). This database is accompanied by a standard search pipeline that facilitates the identification of potential Hg methylators. However, this initiative also highlights the gaps in our understanding of Hg methylators, including their biodiversity, abundance, and environmental significance.

Previous studies have demonstrated that microbial Hg methylation is closely associated with sulfur and nitrogen biogeochemical cycling, as well as with the presence of specific forms of organic matter (OM) (Liu et al. 2014; Moreau et al. 2015; Regnell and Watras 2018; Tisserand et al. 2022; Wang et al. 2022). These associations are likely linked to their influence on the chemical speciation and cellular uptake pathways of Hg(II), which in turn modulate its bioavailability to methylating microorganisms. Several mechanisms have been proposed for Hg(II) uptake by Hg methylators, including the passive diffusion of neutrally charged Hg–S complexes across cell membranes (Drott et al. 2007) and active or facilitated transport via metal transporter systems (Schaefer et al. 2011). While endocytosis-like internalization of HgS nanoparticles is plausible, specific mechanisms in Hg methylators require further investigation. Specifically, reduced sulfur species in the environment can promote the formation of bioavailable Hg(II)–sulfide or Hg(II)–polysulfide complexes, which are more readily taken up by methylating cells (Hsu-Kim et al. 2013; Wang et al. 2019). Similarly, organic matter—especially in particulate form—can affect Hg speciation through complexation reactions and provide microenvironments that enhance Hg(II) bioavailability (Gascón Díez et al. 2016). The recent work has also shown that particulate organic nitrogen may contribute to Hg methylation by serving as a nutrient source that stimulates microbial activity and Hg-transforming populations (Starr et al. 2022), although its direct role in Hg(II) uptake remains to be clarified. A large number of studies found that Hg methylation was carried out in anoxic environments by anaerobic microorganisms (Ma et al. 2019; Rosati et al. 2018). Nonetheless, emerging evidence has highlighted the potential for MeHg formation in water columns characterized by low-oxygen levels (Cabrol et al. 2023; Gionfriddo et al. 2016; Lamborg et al. 2014, 2008; Lin et al. 2021; Wang et al. 2021), although the primary location (suboxic or anoxic layers) of MeHg production remains a controversial issue. In addition, sinking particles have been verified to provide anoxic microenvironments in oxic and low-oxygen water columns, expanding the habitat for Hg methylators and serving as hotspots for Hg methylation (Capo et al. 2020, 2023; Ortiz et al. 2015; Wang et al. 2021).

Marine low-oxygen environments have been considered critical habitats for various microorganisms (Breitburg et al. 2018). Over the decades, many studies have focused on low-oxygen environments such as the Gulf of Mexico (Rabalais et al. 2002), Black Sea (Murray et al. 1989), Saanich Inlet located in Vancouver Island, British Columbia, Canada (Hawley et al. 2017), and the oxygen minimum zones (OMZs) in the eastern tropical Atlantic and Pacific Oceans (Karstensen et al. 2008). These studies have demonstrated a profound contribution from microorganisms in low-oxygen environments to carbon cycling (Enge et al. 2016), sulfur and nitrogen cycling (Canfield et al. 2010; Suter et al. 2021), arsenic respiration (Saunders et al. 2019), and anaerobic methane oxidation (Steinsdóttir et al. 2022).

As one unusual example of low-oxygen ecosystem, marine blue holes are essentially limestone caves formed by karst processes (dissolution and/or conduit collapse) during the last glacial period and usually exist at 100–200 m below current sea level (Martin et al. 2012; Xie et al. 2019; Yao et al. 2020). Blue holes have little photosynthetic oxygen production and limited vertical mixing, resulting in marine oxygen gradients and biogeochemical hotspots (Kindler et al. 2021). Within these specialized niches, a multitude of diverse microbial lineages thrive, each playing a pivotal role in the unique ecological dynamics (Doni et al. 2024; Patin et al. 2021). The Yongle Blue Hole (YBH) in the South China Sea has been recognized as the world's deepest blue hole with a depth of 301 m, almost 100 m deeper than the second deepest blue hole – Dean’s Blue Hole in Long Island, Bahamas (Gonzalez et al. 2011). YBH features a sharp redox cline with anoxic and sulfidic bottom seawater (Chen et al. 2023a, b; He et al. 2020; Sun et al. 2023), suggesting a potential habitat for Hg methylators. For this reason, we hypothesized that (1) YBH contains hotspots for Hg methylation; (2) the distribution of Hg methylators in YBH varies along the redox cline; and (3) the lifestyles of microorganisms may play important roles in Hg methylation.

To test these hypotheses, we conducted metagenomic analyses to study the potential for Hg methylation in YBH. Metagenome-assembled genomes (MAGs) were recovered to investigate the genomic traits and phylogenetics of putative Hg methylators. Relative abundance of the *hgcA* gene was calculated along depth, and Hg methylator relative abundance in free-living (FL) versus particle-associated (PA) samples was also assessed to study the distribution and habitat of putative Hg methylators. Geochemical parameters were measured with depth and tested for correlation with relative abundance of *hgcA* genes. In this study, we aim to shed light on the distribution of Hg-methylating potential in YBH and highlight an important role for blue hole ecosystems in the marine Hg cycle.

## Materials and methods

### Hydrochemical analyses

**Fig. 1:**
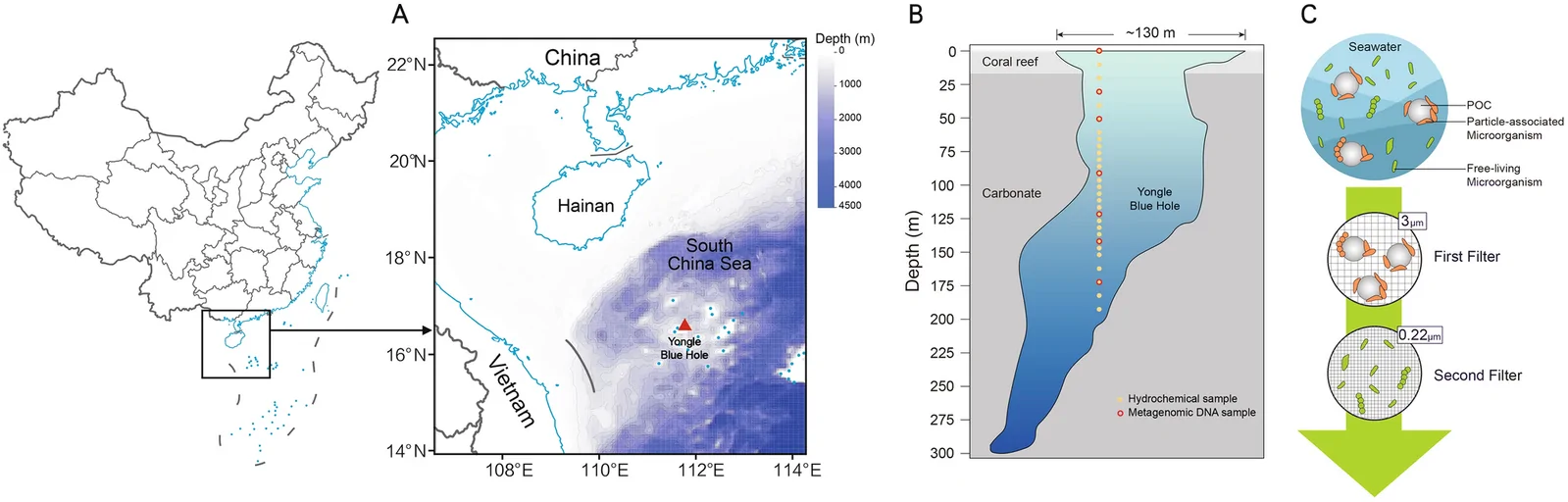
Map of the Yongle Blue Hole sampling site and illustration of sampling methods. **A** The location of Yongle Blue Hole is shown by a red triangle. Bathymetric depth data were obtained from the ETOPO1 database hosted by the NOAA plotted by marmap R package (Pante and Simon-Bouhet 2013). **B** Samples collected in this study and vertical profile of Yongle Blue Hole modified from (Li et al. 2018). Samples for hydrochemical analyses and metagenomic sequencing are shown by yellow solid circles and red hollow circles, respectively. **C** Method of obtaining free-living and particle-associated microorganisms separately by filtration through membranes of two different pore sizes

The methodologies for the seawater sampling and physiochemical analyses have been previously described in detail (Chen et al. 2023a, b; Sun et al. 2023). Briefly, seawater samples from 29 different depths of YBH (111.768° E, 16.525° N, Fig. 1A) from 0 to 190 m were collected to measure hydrological parameters (Fig. 1B). The concentration of dissolved oxygen (DO) was determined according to Hansen (2009) after the sample was fixed by 2 mol L^−1^ MnCl_2_ and 1 mol L^−1^ NaOH/KI, with a detection limit of ~ 0.3 μmol L^−1^. Sulfide samples collected from the Niskin bottles were immediately fixed in situ with zinc acetate. The total sulfide species concentration (referred to as H_2_S) was measured in an onshore laboratory using a spectrophotometer at 670 nm after color development (Cline 1969), which has a detection limit of ~ 3 μmol L^−1^). For dissolved organic carbon (DOC) analysis, samples were filtered through pre-combusted glass-fibre filters and the filtrates were collected and stored at -20°C. Concentrations of DOC were measured by high-temperature catalytic oxidation (Sugimura and Suzuki 1988) using a Total Organic Carbon analyzer (Shimadzu, Japan).

### Metagenomic sample collection

Seawater samples (50 L) from seven depths (0, 30, 50, 90, 120, 140, 170 m) of YBH were collected in October 2019 from an anchored working platform as previously described (Xie et al. 2019). These samples were collected at specified depths using 12 L Niskin bottles. To obtain a sufficient volume for metagenomic analyses, water from multiple Niskin bottles was combined at each depth. The samples were then sequentially filtered through a 3 μm and a 0.22 μm polycarbonate film (Millipore ®) successively. The microorganisms retained on the 3 μm membrane were regarded as PA microbes, while the microorganisms passed through the 3 μm membrane but retained on the 0.22 μm membrane were regarded as FL microbes (Fig. 1C). Both membranes were preserved in liquid nitrogen and subsequently transferred to a − 80 °C freezer in the laboratory until DNA extraction.

### DNA extraction and sequencing

The protocol used for metagenomic DNA extraction was modified from Zhou et al. (1996). Briefly, the membrane filter was washed with an extraction buffer [100 mM Tris–HCl (pH 8.0), 100 mmol L^−1^ EDTA Na (pH 8.0), 100 mmol L^−1^ sodium phosphate (pH 8.0), 1.5 M NaCl, 1% (w/v) CTAB], followed by centrifugation at 5000×*g* for 20 min (25 °C). The pellets were ground in liquid nitrogen and digested with proteinase K at 37 °C and then incubated with SDS at 65 °C for 30 min. The DNA was then extracted with phenol/chloroform, and precipitated by adding 0.6 volumes of isopropanol, followed by a wash with 70% (v/v) alcohol and finally air-dried. The resulting DNA was dissolved and stored in TE buffer. The concentration and integrity of DNA were determined by Qubit and agarose gel electrophoresis. The sequencing of a total of 14 DNA samples was performed at BGI (Shenzhen, China) on the Illumina HiSeq X-Ten platform to produce 2 × 150 bp paired-end reads.

### Metagenome assembly and binning

All metagenomic reads were processed using Trimmomatic v0.38 (Bolger et al. 2014) to remove adapter sequences and low-quality reads. Two strategies were used for assembly and binning. First, every sample was assembled separately by metaSPAdes v3.15.4 (Bankevich et al. 2012) with default parameters, then contigs were binned using VAMB v3.0.5 (Nissen et al. 2021) with an unsupervised machine learning approach. Second, the 14 samples were divided into three groups based on the redox potential at their sampling depths, following the methodology of a previous study on the same site in the YBH (Zhang et al. 2021). Specifically, samples collected from 0–50 m, 60–100 m, and 105–190 m were classified as “surface”, “intermediate”, and “deep” waters, respectively. Each of these three groups was then further classified into two sub-groups according to microbial cell “lifestyles”, *i.e.*, free-living or particle-associated. Quality-filtered short reads from each sub-group were merged and assembled with MEGAHIT v1.2.9 (Liu et al. 2015) using the ‘–presets meta-sensitive’ parameter to optimize assembly for metagenomic data. MAGs were recovered using metaBAT (Kang et al. 2015), metaBAT 2 (Kang et al. 2019), and MaxBin (Wu et al. 2014). MAGs from the three binning tools were merged and refined using metaWRAP v1.3 (Uritskiy et al. 2018). Finally, MAGs derived from both strategies were combined and dereplicated using dRep v3.0.0 (Olm et al. 2017) with default parameters. CheckM v1.1.11 (Parks et al. 2015) was used to determine the quality of MAGs. Only high-quality MAGs (completeness > 75%, contamination < 5%) were retained for downstream analyses. To determine the taxonomy of the MAGs, 16S rRNA genes were predicted by RNAmmer v1.2 (Lagesen et al. 2007) and BLASTed against the NCBI-nt database. For MAGs lacking 16S rRNA genes, their taxonomy was determined based on the concatenated protein phylogeny using GTDB-Tk v1.7.0 (Parks et al. 2018) with the GTDB r202 release. The GTDB taxonomy assignments were then converted to the corresponding NCBI taxonomy using the built-in script provided with GTDB-Tk. Prokka v1.14 (Seemann 2014) was used to predict and annotate genes. All amino acid sequences were searched against the KEGG Pathway Database using BlastKOALA (http://www.kegg.jp/blastkoala/), and completeness of pathways was estimated by KEGG-Decoder (Graham et al. 2018).

### *hgcA* genes prediction

The *hgcA* hmm profile provided in the Hg-MATE database v1.01142021 (Capo et al. 2022) was used to search for *hgcA* genes by HMMER v3.3.2 (Finn et al. 2011) with the parameter “–incE 1E-25”, as recommended by the database protocol. The conserved motif [N(V/I)WC(A/S)(A/G)GK] was used to further verify genuine HgcA sequences. Putative sequences shorter than 100 amino acids were discarded. To remove redundancy, duplicated HgcA sequences were clustered using CD-HIT v4.8.1 (Godzik and Li 2006) with the parameters “-c 1 -n 5”, and only one representative sequence from each cluster was retained. The taxonomy of HgcA sequences in MAGs was inferred according to their host MAGs. For the remaining HgcA sequences, taxonomy was predicted using BLASTp against the NCBI-nr database.

### Phylogenetic analysis

All HgcA amino acid sequences derived from this analysis were aligned with MAFFT v7.490 (Standley and Katoh 2013). A maximum likelihood (ML) tree of HgcA sequences was reconstructed with IQ-TREE v2.0.3 (Schmidt et al. 2014). The best-fit model LG + F + R6 was selected automatically by IQ-TREE. Three CO dehydrogenase/acetyl-CoA synthase subunit delta (CdhD) sequences from the genus *Methanosarcina* (Q8PRQ5.1), *Archaeoglobus* (O29870.1), and *Dehalococcoides* (AAW40003.1) were retrieved from GenBank and were used as outgroups to reroot the HgcA tree using the web-based phylogenetic tree editor iTOL (Letunic and Bork 2021). To determine the phylogenetic placement of the HgcA sequences identified in YBH relative to the HgcA sequences available in public databases, a reference dataset of 185 HgcA sequences was retrieved from the Hg-MATE database, representing diverse taxonomic groups. These reference sequences were combined with the HgcA sequences obtained in this study, and a phylogenetic tree was constructed using the LG + C50 best-fit model, following a similar procedure as described above.

### Structure prediction

To further validate the functionality of the putative HgcA sequences identified in YBH, twelve representative HgcA sequences were selected for computational structural analysis. Specifically, all predicted HgcA protein sequences derived from MAGs were clustered using CD-HIT (Godzik and Li 2006) at a 50% sequence identity threshold, and the representative sequences from each cluster were selected by default settings. Protein structure prediction was then performed using AlphaFold 3 (Abramson et al. 2024). The predicted local distance difference test (pLDDT) score was used to estimate the per-residue model confidence, while the predicted template modeling (pTM) score assessed the overall structural accuracy of the models. Structural comparisons were conducted using UCSF ChimeraX v1.9. The ‘matchmaker’ tool was employed to align each predicted YBH HgcA model to the reference structure of HgcA (PDB ID: 9A0B) reported by Cooper et al. (2020), which was derived through coevolution-based modeling. Root mean square deviation (RMSD) values were calculated following the pruning of outlier atom pairs to assess structural similarity within the globular domain.

### Relative abundance calculation

All metagenomic reads were mapped to predicted *hgcA* genes using Minimap2 (Li 2021), and the coverage depth normalized by gene length was determined with the jgi_summarize_bam_contig_depths script implemented in the MetaBAT2 software (Kang et al. 2019). MicrobeCensus v1.1.1 (Nayfach and Pollard 2015) was used to estimate the microbial genome count (genome equivalent) of each metagenomic sample. The relative abundance of each *hgcA* gene was then calculated as ‘*hgcA* coverage/genome equivalent × 100%’, representing the percentage of genomes in the metagenome that contain *hgcA* genes.

## Results

### Geochemical features of YBH

The general geochemical characteristics of YBH have been reported in recent publications (Chen et al. 2023a, b; Sun et al. 2023). Hence, only pertinent chemical features were described in this study to provide context for the subsequent analyses of Hg methylation. YBH was found to be a natural redox gradient, with DO concentrations nearly saturated (191.98 μmol L^−1^) in the surface water, decreasing to 78.00 μmol L^−1^ at a depth of 50 m. A suboxic layer was formed in the intermediate water from 50 to 100 m depth, where the DO concentration dropped to only 8.06 μmol L^−1^. The water column below 100 m depth was an anoxic environment, with dissolved oxygen concentrations falling below the detection limit. (Fig. S1A). Sulfide was undetectable above 90 m depth, and it remained at a low concentration until 130 m depth, where it began to increase linearly, reaching 249.50 μmol L^−1^ at a depth of 150 m and maintaining this value below that depth (Fig. S1B). The sulfate concentration was 29.45 μmol L^−1^ in the surface water and showed a downward trend with increasing depth, with a concentration of 25.99 μmol L^−1^ detected at 190 m depth (Fig. S1C). The concentration of DOC was 90.75 μmol L^−1^ at the surface, decreasing to 17.63 μmol L^−1^ at 100 m depth, and remained constant below that depth (Fig. S1D).

### MAGs recovered from YBH

In total, 421 MAGs were recovered from the metaWRAP pipeline, whereas 398 MAGs were recovered from the VAMB pipeline. After dereplication of all the MAGs mentioned above, 223 high-quality MAGs with estimated completeness > 75% and contamination < 5% were derived, comprising 202 bacterial MAGs and 21 archaeal MAGs. Eight MAGs were classified as putative new phyla and 37 MAGs were classified as uncultivated candidate phyla according to GTDB-Tk (Table S1). Except for the putative new phyla MAGs, other MAGs belonged to 27 different phyla.

The largest group of the MAGs belonged to Proteobacteria (78 MAGs), of which 39 were Deltaproteobacteria. Bacteroidetes were the second largest group, consisting of 23 MAGs. Euryarchaeota (10 MAGs) was the major group of the archaeal MAGs.

A total of 38 MAGs were detected as carrying *hgcA* genes. Surprisingly, all the *hgcA*-carrying MAGs belonged to Bacteria and no archaeal *hgcA*-carrying MAGs were found. Eight *hgcA*-carrying high quality MAGs were detected with co-recovery of their 16S rRNA genes. These MAGs belonged to seven different phyla, in which more than half of the *hgcA*-carrying MAGs (24 MAGs) were affiliated to the phylum Proteobacteria, and all of them were Deltaproteobacteria (Table S1).

### Diversity and phylogeny of *hgcA* genes in YBH

Since not all *hgcA* genes were binned into MAGs, additional *hgcA* genes were retrieved from metagenomic contigs. In total, 135 diverse *hgcA* sequences were found in the YBH dataset, including the 38 sequences in the MAGs mentioned above. These 135 sequences, referred to as the 'YBH-HgcA dataset,' were used in subsequent analyses. All HgcA sequences in the YBH-HgcA dataset were predicted to be affiliated with Bacteria, with no archaeal HgcA sequences identified.

**Fig. 2:**
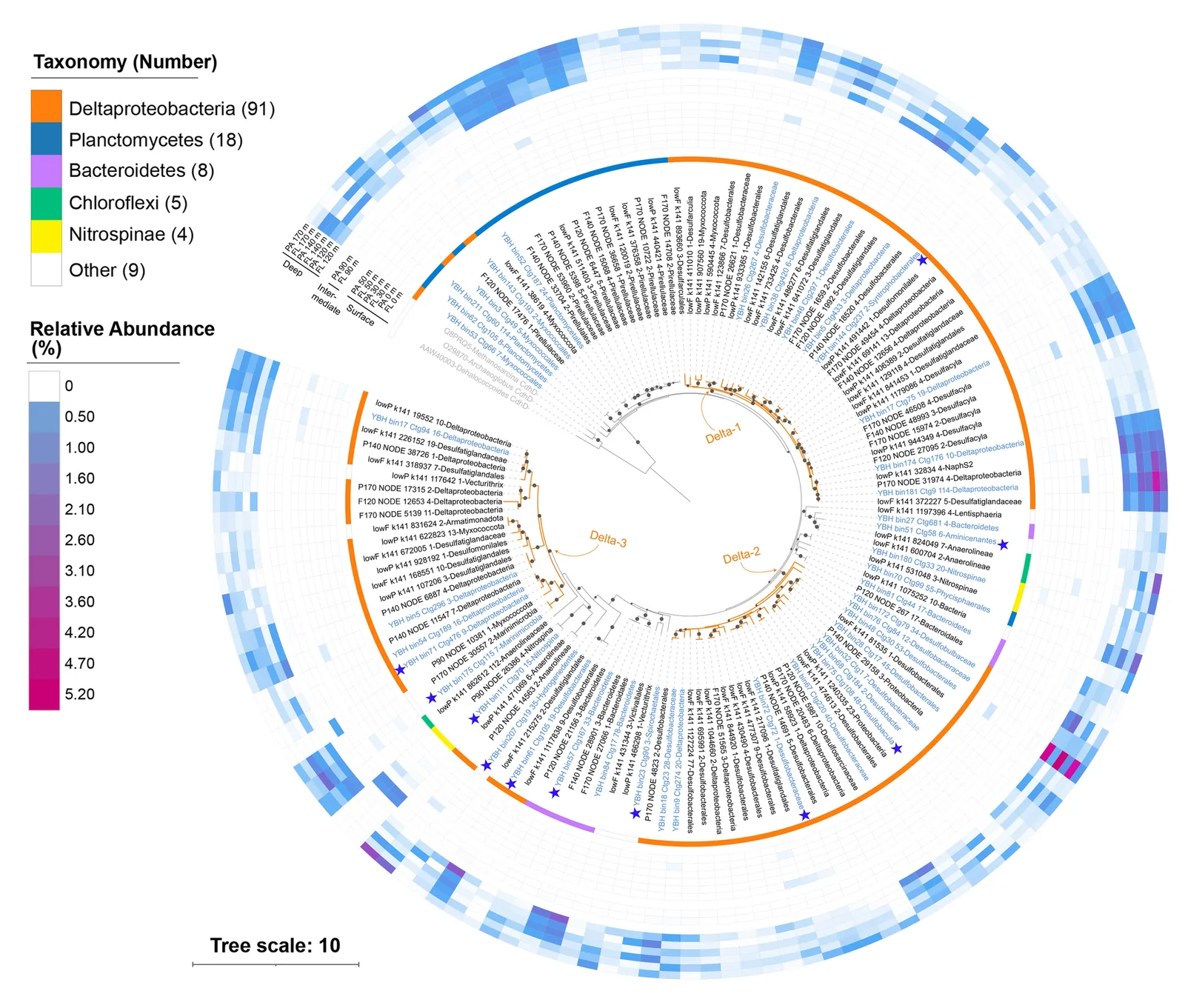
Maximum-likelihood phylogenetic tree of HgcA amino acid sequences (1000 ultrafast bootstrap replicates; values > 90% are shown by black dots at the nodes). HgcA sequences that were binned into MAGs are shown in blue, while other HgcA sequences predicted from the contigs are shown in black. Three CO dehydrogenase/acetyl-CoA synthase subunit delta (CdhD) sequences were used as an outgroup and are shown in gray. Blue stars indicate representative sequences for AlphaFold modelling. Taxonomic classifications of the *hgcA* sequences are labeled in the outer circle by different colors. The three deltaproteobacterial clades (Delta-1, Delta-2, and Delta-3) discussed in the text are highlighted by orange branches. The relative abundance of the *hgcA* genes is shown by the heatmap. Scale bar indicates substitutions per site

In the YBH-HgcA dataset, ninety-one HgcA sequences were found to be affiliated with Deltaproteobacteria, comprising more than 60% of the YBH-HgcA dataset. Other HgcA sequences were affiliated with Planctomycetes (18), Bacteroidetes (8), Chloroflexi (5), Nitrospinae (4), and a few other groups. Based on the ML tree of the YBH-HgcA dataset, Deltaproteobacteria-HgcA sequences showed high diversity and formed three different main clusters, designated as “Delta-1”, “Delta-2”, and “Delta-3”, respectively, as shown in Fig. 2. The Delta-1 clade contained 38 nonredundant Deltaproteobacteria-HgcA sequences, forming the largest cluster in the tree. The Delta-3 clade exhibited the longest branch length and included a few members from other phyla, suggesting greater evolutionary divergence compared to other HgcA sequences. Most Planctomycetes-HgcA were affiliated with the family Pirellulaceae and clustered together, while several other Planctomycetes-HgcA sequences were clustered with Myxococcales-HgcA, forming several small clusters near the root. Most Bacteroidetes-HgcA sequences were clustered into a single cluster. Chloroflexi- and Nitrospinae-associated HgcA sequences fell into two separate clusters, respectively (Fig. 2).

To assess the novelty of the HgcA sequences identified in the YBH, all HgcA sequences obtained in this study were combined with representative sequences from the Hg-MATE database to construct an expanded HgcA phylogenetic tree **(**Fig. S2). In this expanded tree, the Deltaproteobacteria-HgcA sequences from the YBH-HgcA dataset still formed three distinct clusters, with a few independent sub-clusters present. In addition, the Pirellulaceae-HgcA sequences formed a monophyletic group, closely related to several Aminicenantes and Elusimirobia sequences from the Hg-MATE database. The Delta-3 clade still exhibited the longest branch length in the expanded tree, with some neighbors from Nirospina, Chloroflexi, and Heimdallarchaeota, indicating more genetic modifications and a complex evolutionary history for this clade.

### AlphaFold models of HgcA sequences

The functionality of the putative HgcA sequences identified in YBH was further evaluated by comparing them with experimentally confirmed HgcA sequences using multiple sequence alignment and protein structure modeling. Twelve representative HgcA sequences were selected using CD-HIT for this analysis. All of these YBH-derived HgcA sequences contained the conserved [N(V/I)WC(A/S)(A/G)GK] motif (Fig. S3), which forms a "cap-helix" structure in the globular domain and harbors a strictly conserved cysteine residue capable of binding the cobalt from a cobalamin ligand and facilitating methyl group transfer to mercury (Fig. S4). The modeled structures also revealed that these HgcA proteins possess a transmembrane region composed of five helices, consistent with previously reported HgcA structures (Cooper et al. 2020; Parks et al. 2013). Although certain regions of the AlphaFold3 models exhibited relatively low pLDDT confidence scores, likely due to sequence novelty or intrinsically disordered regions complicating precise structure prediction, all models demonstrated overall high pTM scores (≥ 0.73). Importantly, the functionally relevant regions, including globular domains and transmembrane segments, consistently showed high local structural confidence. All twelve YBH-derived HgcA models exhibited notable structural similarity to the reference template 9A0B within the globular domain, with RMSD values ranging from 1.01 to 1.52 Å across 30 to 84 pruned Cα atom pairs (Figure S5). The lowest RMSD was observed in the YBH_bin61-Desulfobacterales model (1.01 Å over 55 atoms), suggesting a high degree of structural conservation in regions associated with cobalamin binding and methyl transfer activity. In contrast, greater structural divergence was noted in the transmembrane helices of the HgcA models. These differences may reflect lineage-specific adaptations of HgcA to distinct membrane compositions or fluidities among different taxa. Such variation in the membrane-anchoring region could influence the localization, orientation, or interaction of HgcA with other cellular components critical for Hg methylation. Notably, the predicted HgcA model from Hydrogenedentes featured an additional globular domain absent from the 9A0B reference structure and other YBH HgcA models (Figure S4). This distinct structural domain, located between the transmembrane helices and the canonical globular region, comprises five β-strands and six α-helices. Its spatial position suggests it may serve as a bridging module, potentially modulating the spatial conformation or accessibility of the catalytic core. Such an insertion could alter the interaction dynamics between HgcA and its cobalamin cofactor or the associated HgcB protein, thus impacting mercury methylation efficiency.

### Abundance of *hgcA* genes in YBH

**Fig. 3:**
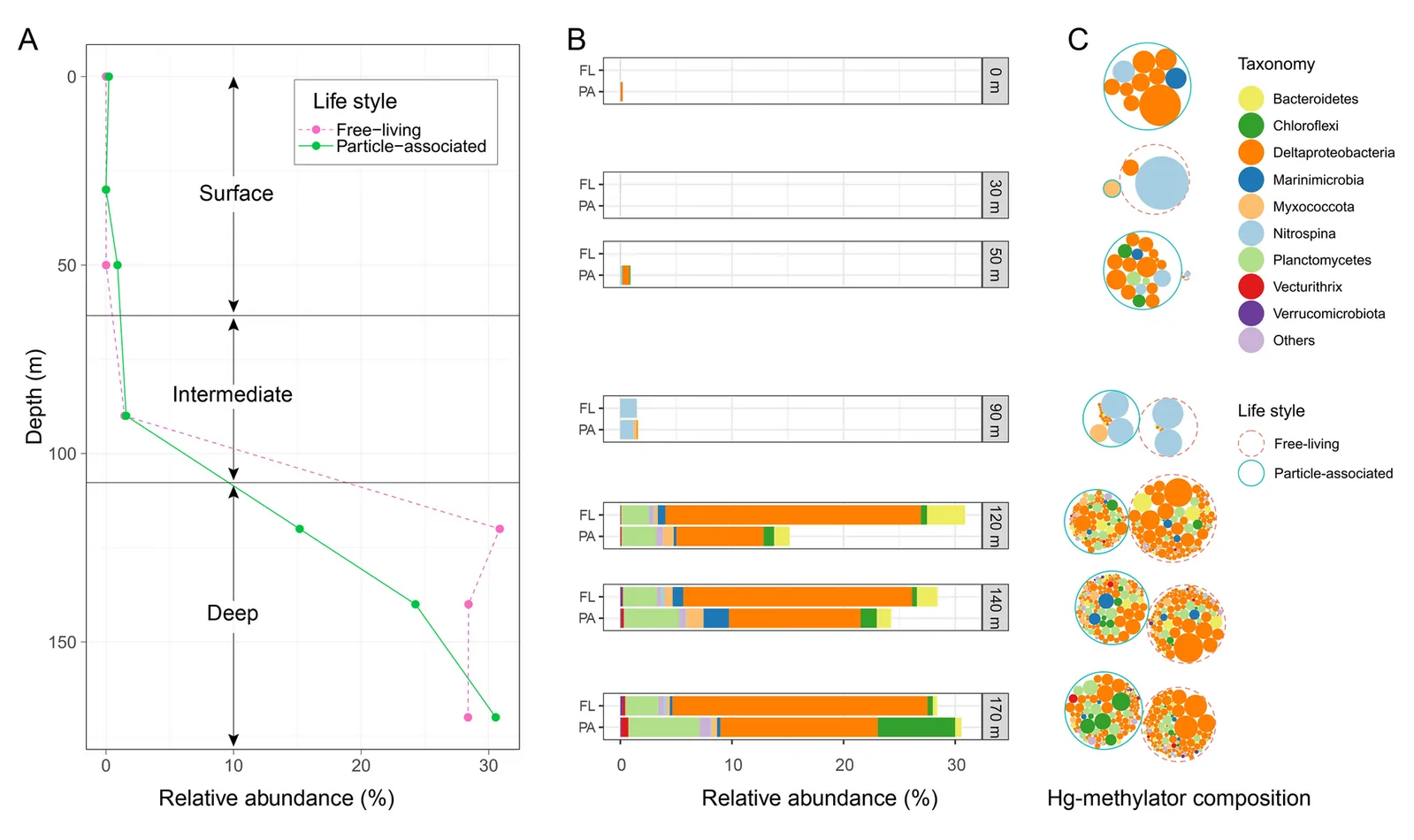
The relative abundance of *hgcA*-carrying methylators. **A** Percentage of *hgcA*-carrying methylators accounting for the YBH microbial community along the depth. The relative abundance in free-living samples and particle-associated samples are represented by red dotted line and green solid line, respectively. **B** Stacked bar plot showing the relative abundance of Hg methylators across various sample depths and lifestyles. Different colors denote distinct taxonomic groups, as indicated in the legend on the right. **C** Hg-methylator composition at different depths. Each *hgcA* homologue is shown by a filled circle, colored by taxonomy. Circle size represents the relative abundance of the *hgcA* homologue within each depth. The total abundance of *hgcA* homologues is scaled to the same value for every depth for better visualization, resulting in the size of the *hgcA* homologue circles comparable in the same depth but not across different depths. *hgcA* sequences from free-living samples or particle-associated samples are involved in red dotted circles or green solid circles, respectively

The oxic surface water samples (0–70 m) contained only a small proportion of *hgcA* genes (Fig. 3A). Specifically, no reads were found in FL samples to be affiliated with *hgcA* genes at 0 m, while only 0.01% of the microorganisms were found to carry *hgcA* genes at 30 m and 50 m depths. PA samples contained a higher proportion of *hgcA*-carrying microorganisms in the surface water. We found that 0.22% of the microbial community might carry *hgcA* genes at 0 m, while the percentage dropped to only 0.001% at 30 m but increased to 0.90% at 50 m depth. In the two low-oxygen intermediate (90 m) water samples, the percentage of *hgcA*-carrying microorganisms increased to 1.46% and 1.58% of the microbial community in the FL and PA samples, respectively.

Notably, a large number of *hgcA* genes were found to occur in the anoxic deep (120–170 m) water. The *hgcA*-carrying microorganisms were estimated to occupy ~ 30% (30.87%, 28.42%, and 28.38% in the 120 m, 140 m, and 170 m samples, respectively) of the microbial community in the three FL samples, while their relative abundance in the three PA samples increased along depth (15.18%, 24.26%, and 30.55% in the 120 m, 140 m, and 170 m samples, respectively). Throughout the entire water column of the YBH waters, the abundance of the *hgcA* gene in PA samples exhibited higher levels at depths of 0 m, 50 m, 90 m, and 170 m. In contrast, FL samples displayed greater *hgcA* gene abundance at depths of 30 m, 120 m, and 140 m.

### Habitat preference of different putative Hg methylators

Deltaproteobacteria were predicted to be the dominant Hg-methylating group in YBH at nearly all depths except for 30 m and 90 m (Fig. 3B, C), in which the Deltaproteobacterial MAG YBH_bin48 was the most abundant *hgcA*-carrying microorganism, accounting for 4.31%, 5.16% and 3.85% of the microbial community in the FL samples at 120 m, 140 m, and 170 m depths. Intriguingly, this MAG only occupied no more than 1% at the same depths as above in the corresponding PA samples, and it was almost undetectable at 0–90 m depths. The same distribution pattern could be observed for many other *hgcA*-carrying microorganisms in different taxonomies (Fig. 2).

Meanwhile, the *hgcA*-carrying microorganisms that exhibited higher abundance in PA samples could be also seen. For example, the *hgcA* sequence ‘lowP_k141_471069_6-Anaerolineae’ affiliated with Anaerolineae (Chloroflexi) was predicted to be the most abundant *hgcA* gene in the PA sample at 170 m depth, accounting for 2.36% of the microbial community, but it only occupied 0.07% in the 170 m FL sample.

Although most *hgcA*-carrying microorganisms were enriched in the deep water, several *hgcA*-carriers, like some *Nitrospina* and Myxococcota lineages, were only observed in surface and intermediate water samples, and these latter groups were the dominant Hg methylators in the micro-oxic intermediate water (Fig. 3B, C). In particular, *Nitrospina* was the dominant Hg methylator in 30 m and 90 m samples, and appeared to live in both FL and PA samples.

### Genome content of *hgcA*-carrying MAGs

**Fig. 4:**
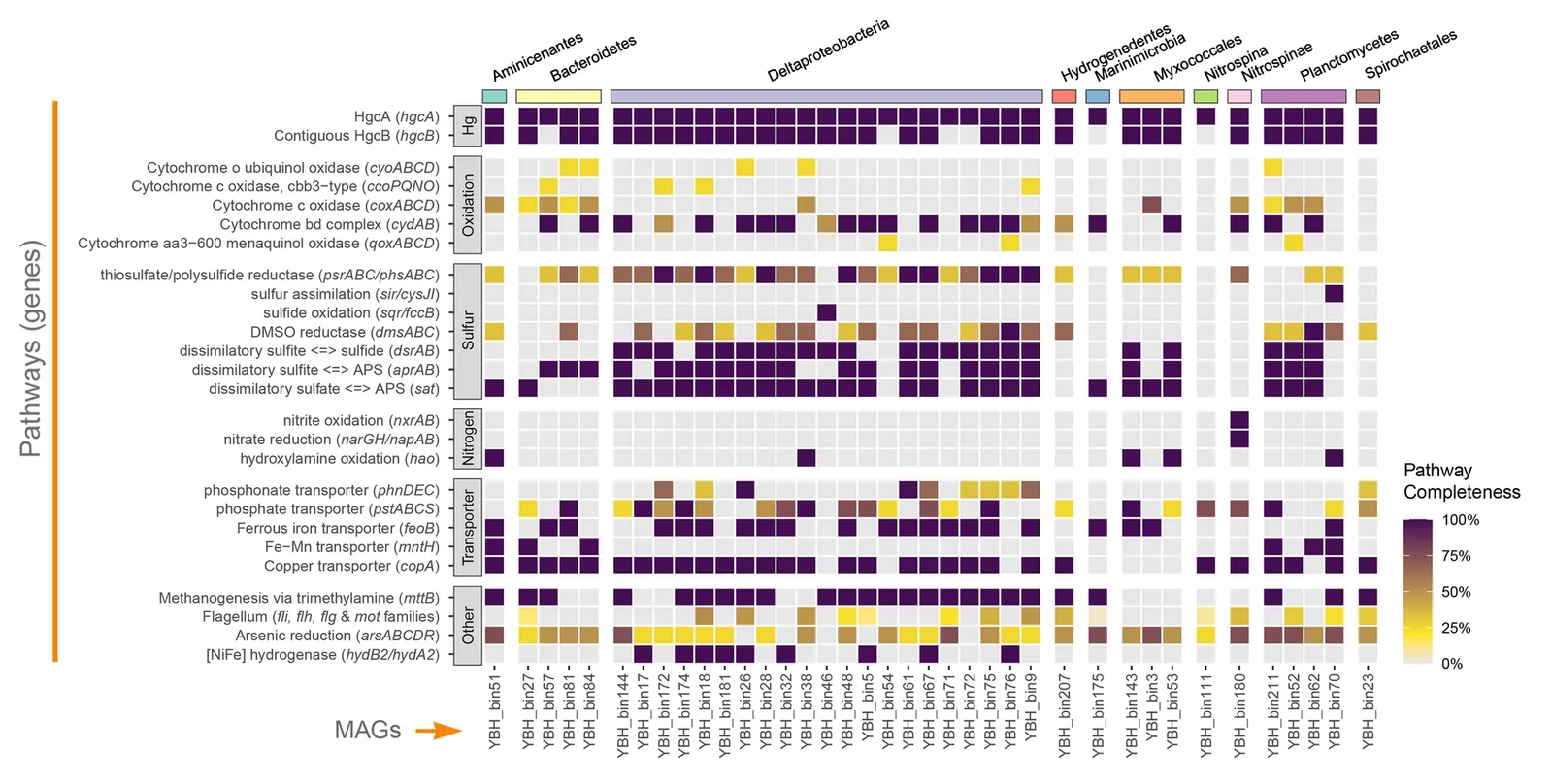
Genome contents of the 38 *hgcA*-carrying MAGs. KEGG pathways are represented on the left side of the heatmap, and the corresponding genes involved in each pathway are exhibited in parentheses. MAGs are represented at the bottom, and their taxonomic classifications are represented at the top by arbitrary colors. Cell colors indicate the completeness of enzymes involved in each pathway according to KEGG-decoder; absent pathways in the MAGs are shown in gray

The genome content and metabolic potential of the 38 *hgcA*-carrying MAGs were studied by mapping the genes to KEGG database (Fig. 4). Most MAGs also carried *hgcB* genes downstream of *hgcA* contiguously, while six MAGs did not possess contiguous *hgcB* genes. The genomic neighborhoods surrounding the *hgcAB* genes did not harbor any other recognizable genes associated with Hg methylation. Complete operons of cytochrome *bd* complex encoding genes (*cydAB*), functioning in oxidative respiratory chains, were found to be carried by half of the MAGs. These MAGs also contained partial operons involved in other terminal oxidase systems, including cytochrome *c* oxidase, cytochrome *o* ubiquinol oxidase, and cytochrome *aa3-600* menaquinol oxidase. Also, genes related to nitrogen and sulfur cycles were found in these MAGs. For example, five MAGs affiliated to Marinimicrobia, Myxococcales, Aminicenantes, and Deltaproteobacteria carried *hao* genes encoding for hydroxylamine oxidoreductase that transform NH_2_OH to NO_2_^−^, and the Nitrospinae YBH_bin180 carried genes encoding for nitrite oxidation and nitrate reduction simultaneously. Besides, most MAGs also carried sulfate-reducing genes, including *dsrAB* encoding for dissimilatory sulfite reductase, *aprAB* encoding for adenosine 5’-phosphosulfate (APS) reductase, and *sat* encoding for ATP sulfurylase. Transporter-encoding genes for ferrous iron and copper were present in most MAGs, and genes encoding for Fe–Mn transporters were found in Planctomycetes, Bacteroidetes, and Aminicenantes-associated MAGs. Furthermore, *mttB* genes that encode for trimethylamine (TMA) methyltransferase catalyzing methanogenesis were found carried by most of the MAGs, and [NiFe] hydrogenase-encoding genes involved in methanogenesis were found in some Deltaproteobacterial MAGs. In addition, nearly all *hgcA*-carrying MAGs harbored various genes involved in the arsenic resistance system. These included the *arsM* gene encoding arsenite methyltransferase, the *arsC* gene encoding arsenate reductase, the *arsR* gene encoding an arsenic resistance transcriptional regulator, and the *arsA* gene encoding an arsenical pump-driving ATPase responsible for expelling arsenic ions. Finally, genes related to flagellation were found in some of the MAGs, although the operons were not complete.

## Discussion

### Environmental conditions favoring Hg methylation in YBH

Although Hg methylation occurring in low-oxygen environments has attracted the attention of researchers in the recent years, no study has yet assessed the Hg methylation potential in blue hole ecosystems. YBH, as the deepest blue hole in the world with a natural redox gradient, provides an ideal environment in which to study Hg methylation under different redox conditions (Chen et al. 2023a, b; He et al. 2020; Sun et al. 2023). In this study, the DO concentration in YBH decreased to undetectable below a depth of 100 m, forming an anoxic environment (Fig. S1**A**). Similar anoxic conditions have been identified as hotspots for Hg methylators in other studies (Chakraborty et al. 2016; Cossa et al. 2009; Podar et al. 2015). Also, sulfide was found to accumulate below a depth of 90 m and reached 250 μmol L^−1^ at the deep layer of the YBH (Fig. S1**B**), providing a rich sulfidic environment that could promote Hg methylation by forming bioavailable Hg(II)–sulfide complexes (Wang et al. 2019). Previous studies found that DOM plays an important role in Hg methylation, especially in the presence of reduced sulfur (Graham et al. 2012; Zhao et al. 2017). The YBH contained ~ 90 μmol L^−1^ DOC in surface waters and ~ 20 μmol L^−1^ DOC in the intermediate and deep waters, which could enhance Hg methylation as well.

### Distinct Hg methylator assemblages across redox zones

More than 30% of the microbial community were found to potentially methylate Hg in some deep water samples, suggesting that the deep anoxic environment may contribute significantly to marine MeHg production. This result is consistent with our previous study of the Saanich Inlet oxygen minimum zone (Lin et al. 2021), although the dominant methylator communities were distinct between these two marine environments. Deltaproteobacteria was the major group of Hg methylators in the YBH deep water. Intriguingly, we found diverse Deltaproteobacteria contributed to Hg methylation instead of a dominant species (Fig. 2). A diversity of over 90 distinct *hgcA* homologues was identified primarily associated with Deltaproteobacteria. This extensive variety implies a relatively low selective pressure in the YBH ecosystem for specific *hgcA* phylotypes. Many HgcA sequences identified in the YBH-dataset formed distinct and previously unrecognized clusters in the phylogenetic tree when compared to sequences available from public databases, particularly within the Delta-1, Delta-2, and Delta-3 clades, with Delta-3 exhibiting the greatest divergence (Fig. S2). The scattered clustering of Deltaproteobacteria-HgcA in the phylogenetic tree may be explained by extensive gene loss during evolutionary history, possibly due to environmental changes and adaptive evolution, as suggested by previous evolutionary analyses (Lin et al. 2023). This finding also highlights the novelty of the Hg-methylating microbial diversity present in the YBH ecosystem and suggests the significance of the blue hole in Hg methylation research. Notably, while only a small proportion of Hg methylators were observed in the surface and intermediate layers of the YBH, we found that the community composition of the methylators in the surface and intermediate waters was distinct from that of the deep water. *Nitrospina* and Myxococcota were the two major Hg-methylating groups that thrived in the suboxic intermediate layer, in which *Nitrospina* has been reported to be the potential methylator in many suboxic ecosystems (Villar et al. 2020) including the Antarctic sea ice (Gionfriddo et al. 2016), Saanich Inlet (Lin et al. 2021), and the Western North Pacific (Tada et al. 2021). Notably, the intermediate suboxic layer exhibited distinctly lower abundance and diversity of both PA and FL *hgcA* homologues compared to the deeper anoxic layers (Fig. 3C). This pattern could be attributed to the unique environmental conditions within this intermediate layer, characterized by a sharp decline in dissolved oxygen but the absence of fully anoxic, sulfidic conditions that prevail in deeper waters (Fig. S1). Such intermediate suboxic conditions may selectively support fewer specialized taxa capable of Hg methylation, leading to reduced diversity of methylators at this depth. Indeed, taxa such as Nitrospina and Myxococcota, which are known to thrive in low-oxygen niches (Sun et al. 2019; Zou et al. 2024), dominated the Hg-methylating community in the intermediate layer. These microorganisms may possess metabolic adaptations enabling effective competition under micro-oxic conditions, thereby outcompeting other potential methylators less suited to this redox environment.

### Contrasting particle-associated and free-living niches

Although the role of sinking particles in the formation of MeHg has been shown in several previous studies (Bravo et al. 2017; Gascón Díez et al. 2016), metagenomic studies that distinguished PA samples from FL samples with regard to Hg methylation potential are barely seen. We analyzed the difference in putative Hg methylator abundance between the PA and FL samples from the same depth of YBH, and surprisingly found that putative methylators with different living styles (i.e., motile versus sessile) exhibited different distribution patterns (Fig. 3). For example, Anaerolineae were the most abundant methylator group in the PA sample at 170 m depths, accounting for 6.93% of the microbial community, but they only occupied 0.46% in the FL sample at the same depth. This result suggested that sinking particles might provide some microorganisms with adaptive living environments, such as anoxic microenvironments, appropriate pH and sulfur content, and/or modulated Hg availability (Bravo et al. 2017), promoting Hg methylation in the water columns. Although previous studies have shown that YBH contained a lower volume of particulate organic carbon (POC) compared to other water columns like the Baltic Sea (He et al. 2020; Le Moigne et al. 2017), coral detritus and the antecedent carbonate platform in which the YBH formed could provide a continuous POC supply (Yao et al. 2020), providing matter and energy source, and therefore create a sustainable living niche for a group of particular Hg methylators. Based on a previous hydrochemical survey of YBH water, peaks in POC were observed at the surface (0 m depth) and at 90 m depth (He et al. 2020). Also, YBH contains richer particulate nitrogen compared to POC, with higher concentrations observed at depths of 30 m, 90 m, and 210 m (He et al. 2020). This may explain why we observed the higher observed *hgcA* gene abundance in PA samples at depths of 0 m, 90 m, and our deepest sample site at 170 m, compared to FL samples. However, the abundance of Hg-methylating microorganisms is not solely determined by the concentration of particulate carbon or nitrogen, as their distribution is likely to be influenced by multiple environmental factors. Moreover, we found that while the abundance of Hg methylators in the FL samples remained at a steady level (~ 30% of the microbial community) in the deep water, it increased along the depth (from ~ 15% at 120 m to ~ 30% at 170 m depth) in the PA samples. This result was consistent with a previous study (Roth Rosenberg et al. 2021) that showed the community structure of FL samples was more stable than that of PA samples under different environmental conditions, also suggesting that sinking particles played a key role in Hg methylation not only in oxygenated environments but also in anoxic environments.

### Ecological adaptations of Hg methylators

By studying the genome content of YBH putative Hg methylators, we found that many of them carry the genes encoding for terminal enzymes functioning in electron transport chains, and exhibited high affinities for oxygen, especially in low-oxygen environments. Most Hg methylators in YBH were also sulfate-reducers, as genes like *dsrAB*, *aprAB*, and *sat* were found in these MAGs (Fig. 4). The production of sulfide by Hg methylators could promote Hg methylation, especially in the presence of DOM in the environment and bioavailable Hg–S–DOM complex is formed (Zhao et al. 2017). YBH has a sulfidic environment with the presence of DOM in the intermediate and deep water, creating a potential suitable environment for Hg methylation. However, the amount and type (molecular weight, aromaticity, and hydrophobicity) of DOM in YBH remains to be characterized, since they may impact bioavailability of Hg complex and further influence the efficiency of methylation (Graham et al. 2012; Moreau et al. 2015). Additionally, nearly all Hg methylators also carried various genes related to arsenic resistance, indicating potential arsenic contamination in the YBH ecosystem. The completeness of the arsenic resistance system was generally lower in Deltaproteobacterial *hgcA*-carrying MAGs compared to other taxa. This pattern may reflect a trade-off in genomic investment shaped by ecological niche specialization. Deltaproteobacteria were predominantly found in the deep, stable, and sulfidic layers of YBH, where arsenic stress may be minimal or relatively constant, potentially reducing the selective pressure to maintain a fully functional arsenic resistance system. In contrast, other taxa with more complete arsenic resistance pathways may inhabit more dynamic environments with fluctuating arsenic exposure, thereby requiring a broader defense mechanism. Moreover, a recent study reported an ArsR-like gene potentially involved in regulating *hgcAB* transcription in the model Hg-methylating bacterium *Pseudodesulfovibrio mercurii* ND132 (Gionfriddo et al. 2023), the *arsR* genes identified in this study shared only ~ 40% sequence identity and were not located in close genomic proximity to the *hgcAB* genes. These findings suggest that the Hg methylators found in YBH might possess alternative regulatory mechanisms governing mercury methylation. Interestingly, a subset of *hgcA*-carrying MAGs also encoded flagellar assembly genes, suggesting potential motility. In a redox-stratified water column such as YBH, motility could allow free-living Hg methylators to actively navigate toward optimal niches with favorable redox conditions or elevated Hg availability. This may help explain the relatively consistent abundance of *hgcA*-carrying taxa in deep anoxic FL samples. Conversely, non-motile methylators, such as Myxococcales, which lack flagellar assembly genes, are likely adapted to exploit sinking particles for passive access to favorable microhabitats. These contrasting strategies highlight the diverse ecological adaptations of Hg methylators in vertically heterogeneous systems.

In addition to gene content, protein structural features may also contribute to functional differentiation among Hg methylators. Despite the high structural similarity observed in the globular domain of YBH-derived HgcA proteins compared to the 9A0B reference structure, the transmembrane domains exhibited more pronounced variability (Fig. S5). This divergence likely arises from adaptation to differing membrane compositions among taxa, potentially reflecting variation in lipid bilayer architecture, redox gradients, or protein–protein interactions specific to different cellular environments (Dominguez et al. 2016; Pogozheva et al. 2013). Such variability may affect the localization, stability, or efficiency of HgcA anchoring and function. Remarkably, the HgcA model from Hydrogenedentes displayed an additional globular subdomain located between the transmembrane helices and the canonical catalytic core (Fig. S4). This domain was absent in all other models. Its spatial position suggests a possible regulatory or structural role, potentially altering the accessibility or positioning of the cobalamin-binding motif or affecting interaction with HgcB. While the precise function of this novel domain remains unknown, it raises the possibility that such structural elaborations may represent lineage-specific adaptations that modulate HgcA methylation efficiency. Experimental studies, such as mutagenesis or protein–protein interaction assays, are needed to elucidate the functional implications of this structural innovation.

### Limitations and future directions

While this study did not include direct measurements of total Hg and MeHg concentrations, our findings nonetheless provide significant insights into the microbial potential for Hg methylation in the unique ecosystem of the YBH. We recognize that the absence of chemical Hg data limits our ability to directly correlate microbial community composition with actual methylation activity and MeHg levels. However, the primary objective of this investigation was to characterize, for the first time, the genomic potential for Hg methylation in a blue hole system, thereby emphasizing the importance of blue hole ecosystems as model environments for studying microbially mediated Hg cycling in stratified aquatic systems. Future studies that integrate multi-omics analyses with concurrent measurements of total Hg and MeHg concentrations will be essential for establishing explicit links between microbial community structure, functional gene abundance, and mercury methylation processes. Such integrated approaches will enhance our understanding of the environmental controls on Hg biogeochemistry and facilitate broader assessments of the ecological importance of these distinctive marine habitats.

## Conclusion

In summary, our study provides the first detailed metagenomic characterization of microbial Hg methylation potential within the world's deepest blue hole ecosystem. We reveal that Deltaproteobacteria likely dominate MeHg production in the deep, anoxic layers of YBH, highlighting the distinctive composition and complexity of the Hg-methylating community. The observed variability in the distribution patterns of Hg methylators corresponds to different microbial lifestyles and ecological niches, emphasizing the critical role that sinking particles play in facilitating mercury methylation processes. Our findings highlight the unique characteristics of marine blue holes and establish them as valuable model ecosystems for advancing the understanding of microbial mercury cycling across diverse aquatic habitats.

## Supplementary Information

Below is the link to the electronic supplementary material.

Supplementary file1 (PDF 200 KB)

Supplementary file2 (PDF 1133 KB)

Supplementary file3 (PDF 1088 KB)

Supplementary file4 (PDF 21671 KB)

Supplementary file5 (PDF 32033 KB)

Supplementary file6 (XLSX 24 KB)

Supplementary file7 (XLSX 41 KB)

## Data Availability

The metagenomic data used in this study are available in the NCBI GenBank under the accession number PRJNA958613. Phylogenetic trees and custom visualization scripts are available from GitHub repository: https://github.com/SilentGene/YBH-Hg
