# Supplementary material for: Metagenomics reveals diverse community of putative mercury methylators across different biogeochemical niches in Sansha Yongle blue hole
Source: Mar Life Sci Technol. 2025 Nov 19;8(1):206–20. doi: 10.1007/s42995-025-00332-7 (PMC12953829; doi:10.1007/s42995-025-00332-7)
Supplement: Supplementary file 1 — Supplementary file1 (PDF 200 KB) [file 42995_2025_332_MOESM1_ESM.pdf]

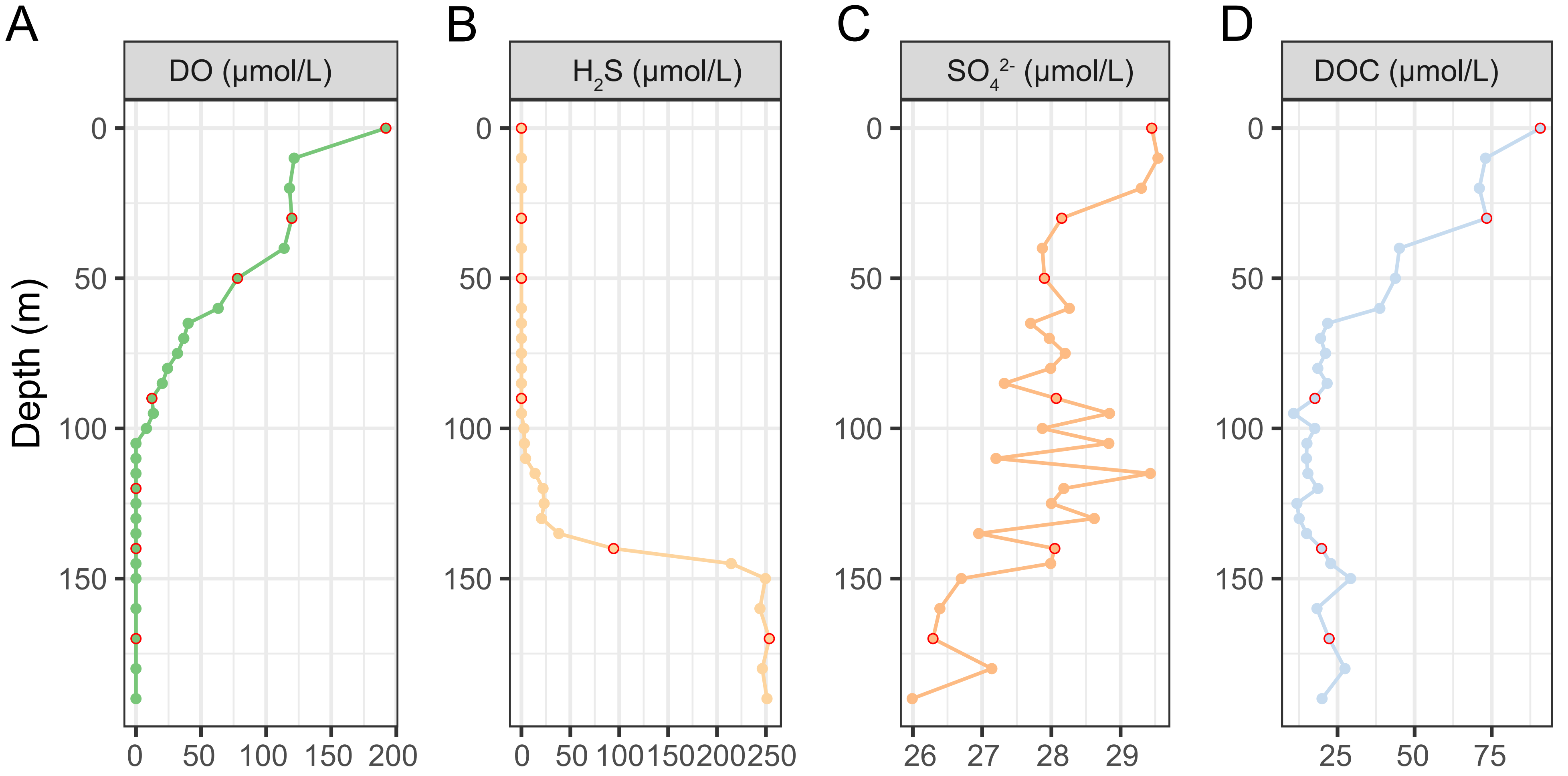

Figure S1. Vertical profiles of hydrochemical characteristics in the Yongle blue hole. The points with red borders in the line graph indicate the locations where corresponding metagenomic sequencing data are available. **(A)** Dissolved oxygen (DO), **(B)** Hydrogen sulfide (H_2S), **(C)** Sulphate (SO_4^{2-}), and **(D)** Dissolved organic carbon (DOC). (Figure adapted from [52])
