# Supplementary material for: Metagenomics reveals diverse community of putative mercury methylators across different biogeochemical niches in Sansha Yongle blue hole
Source: Mar Life Sci Technol. 2025 Nov 19;8(1):206–20. doi: 10.1007/s42995-025-00332-7 (PMC12953829; doi:10.1007/s42995-025-00332-7)
Supplement: Supplementary file 2 — Supplementary file2 (PDF 1133 KB) [file 42995_2025_332_MOESM2_ESM.pdf]

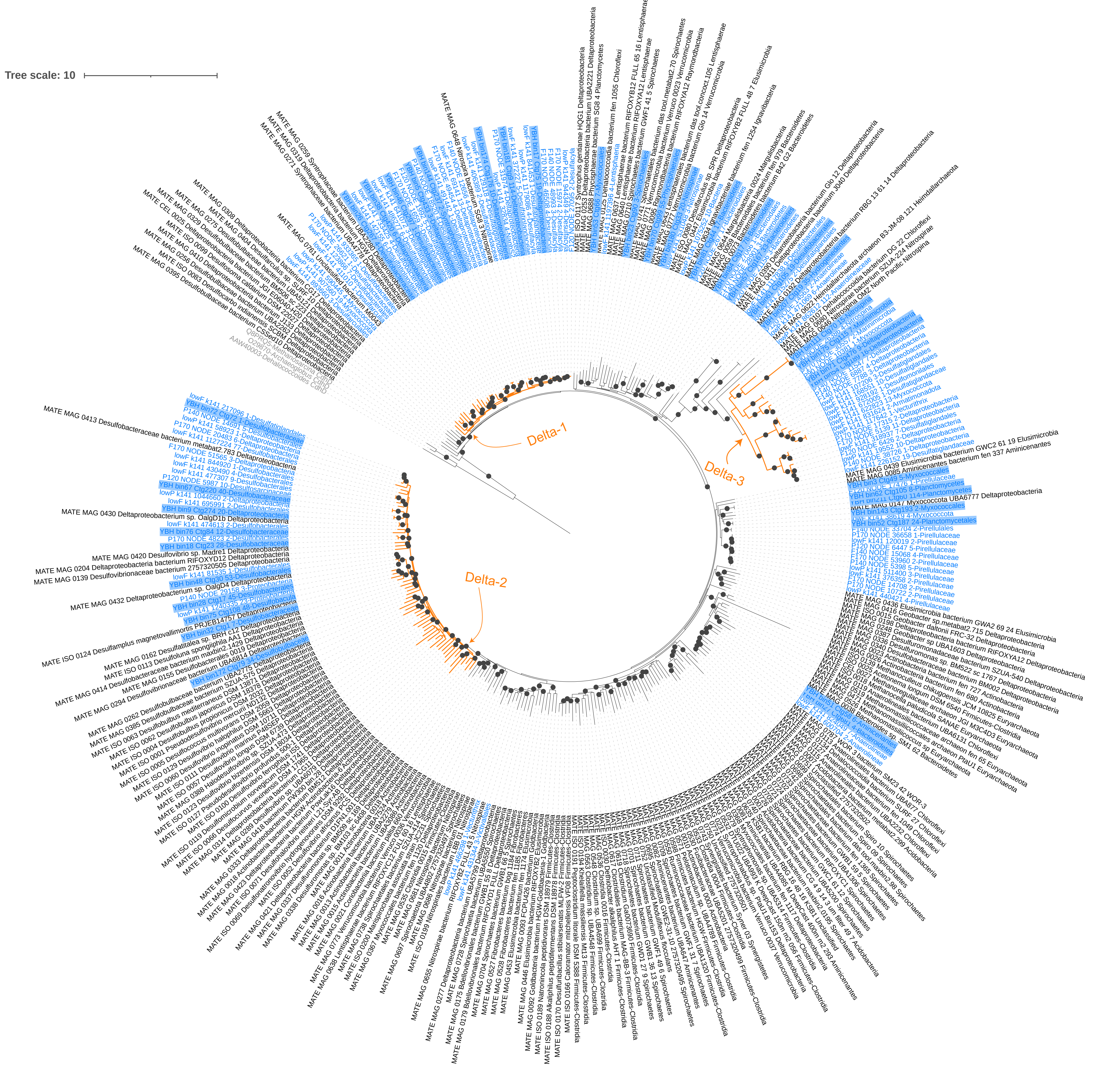

Figure S2. Maximum Likelihood Tree of HgcA Proteins. Sequences obtained in this study are highlighted in blue, with sequences from MAGs having a blue background. Reference sequences from public databases, collected from the Hg-MATE database, are shown in black. Outgroup sequences (CdhD) are represented in gray. The phylogenetic tree was constructed using the L60+C50 amino acid substitution model in IQ-TREE, with 1000 iterations of Ultrafast bootstrap for reliability testing. Branches with Ultrafast bootstrap values > 90 are marked with black dots. The clusters "Delta-1", "Delta-2", and "Delta-3" are defined as in Figure 2 for easier comparison.
