# Supplementary material for: Metagenomics reveals diverse community of putative mercury methylators across different biogeochemical niches in Sansha Yongle blue hole
Source: Mar Life Sci Technol. 2025 Nov 19;8(1):206–20. doi: 10.1007/s42995-025-00332-7 (PMC12953829; doi:10.1007/s42995-025-00332-7)
Supplement: Supplementary file 3 — Supplementary file3 (PDF 1088 KB) [file 42995_2025_332_MOESM3_ESM.pdf]

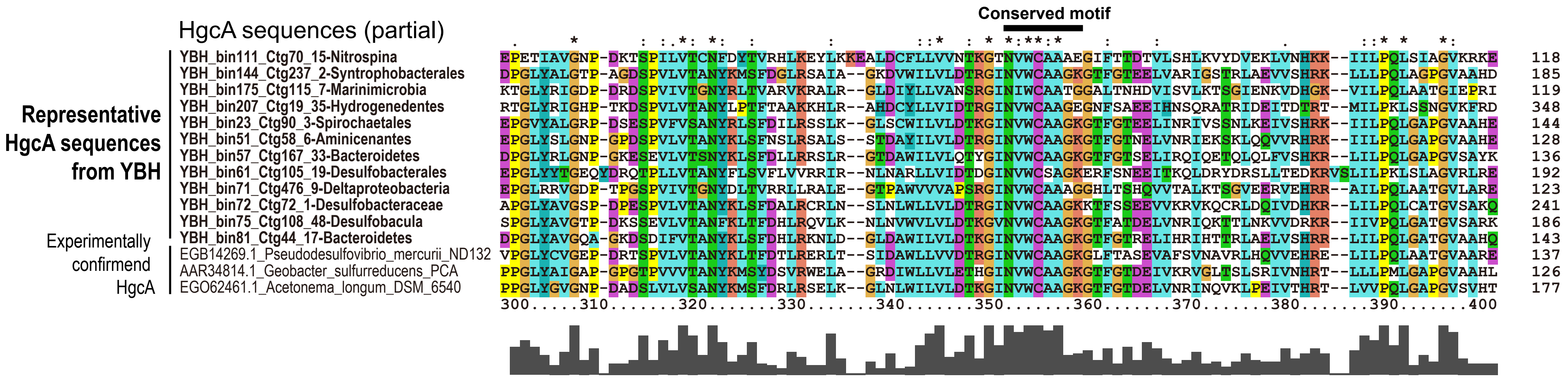

Figure S2. Multiple sequence alignment (MSA) of HgcA sequences. The alignment displays the 12 representative HgcA sequences from this study alongside other experimentally confirmed sequences. Asterisk (*), colon (:) and period (.) symbols indicate respectively identical residues, conserved substitutions and semi-conserved substitutions.The conserved motif of HgcA is labeled. The conservation of each amino acid position is shown below the graph. The numbers on the right side of the MSA figure indicate the position of the last displayed amino acid in each sequence within its original, ungapped sequence. The numbers at the bottom of the MSA figure represent the position information of the entire alignment, accounting for gaps (gapped positions).
