# Supplementary material for: Metagenomics reveals diverse community of putative mercury methylators across different biogeochemical niches in Sansha Yongle blue hole
Source: Mar Life Sci Technol. 2025 Nov 19;8(1):206–20. doi: 10.1007/s42995-025-00332-7 (PMC12953829; doi:10.1007/s42995-025-00332-7)
Supplement: Supplementary file 4 — Supplementary file4 (PDF 21671 KB) [file 42995_2025_332_MOESM4_ESM.pdf]

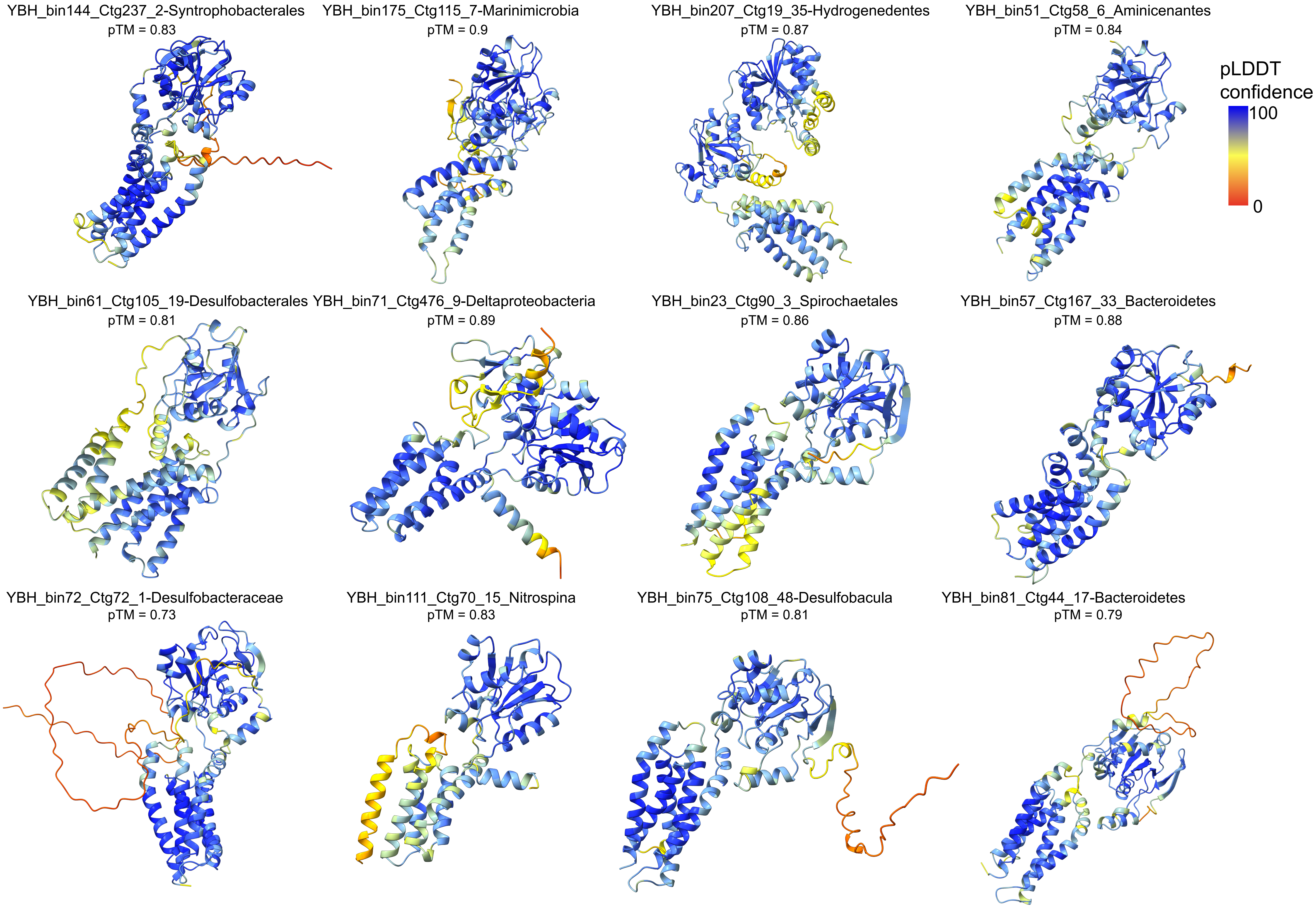

Figure S4. Computational models of representative HgcA proteins generated by AlphaFold3. The models are colored on a blue-to-red scale indicating the pLDDT score for each residue, with red representing low pLDDT and blue representing high pLDDT. The predicted template modeling (pTM) score for each model is also shown to indicate the overall accuracy of the structure.
