# Supplementary material for: Metagenomics reveals diverse community of putative mercury methylators across different biogeochemical niches in Sansha Yongle blue hole
Source: Mar Life Sci Technol. 2025 Nov 19;8(1):206–20. doi: 10.1007/s42995-025-00332-7 (PMC12953829; doi:10.1007/s42995-025-00332-7)
Supplement: Supplementary file 5 — Supplementary file5 (PDF 32033 KB) [file 42995_2025_332_MOESM5_ESM.pdf]

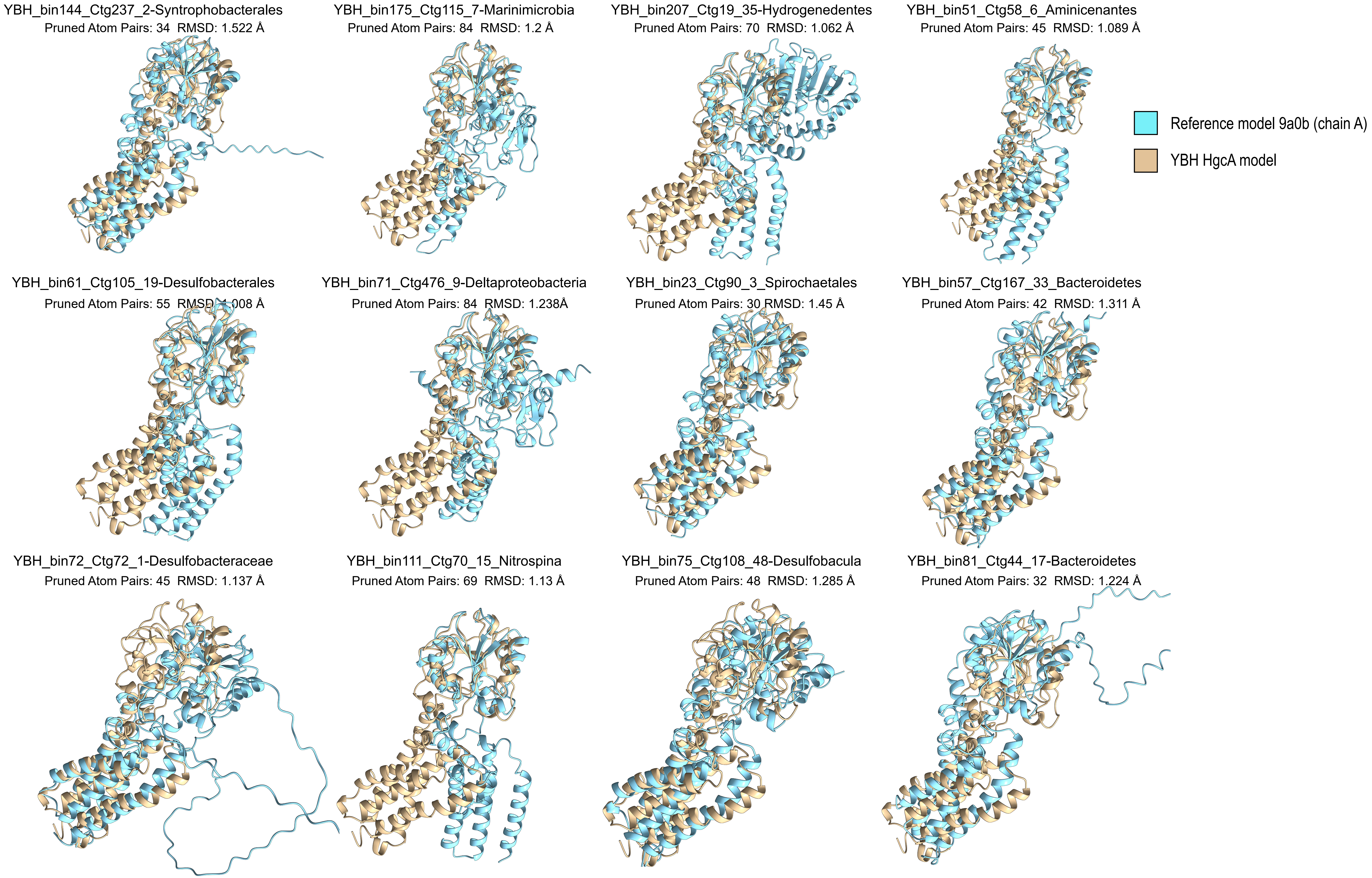

Figure S5. Structural alignment of 12 representative HgcA models with the reference structure (PDB ID: 9a0b, chain A) using ChimeraX MatchMaker. The reference model is shown in cyan, and the YBH HgcA models are displayed in brown. The number of pruned atom pairs and the corresponding RMSD (in Å) are indicated in each panel.
